# Blood Flow Within Bioengineered 3D Printed Vascular Constructs Using the Porcine Model

**DOI:** 10.3389/fcvm.2021.629313

**Published:** 2021-06-07

**Authors:** Nhu Thao N. Galván, Samantha J. Paulsen, Ian S. Kinstlinger, Juan C. Marini, Inka C. Didelija, Dor Yoeli, Bagrat Grigoryan, Jordan S. Miller

**Affiliations:** ^1^Department of Surgery, Baylor College of Medicine, Houston, TX, United States; ^2^Department of Bioengineering, Rice University, Houston, TX, United States; ^3^Department of Pediatrics-Critical Care, Baylor College of Medicine, Houston, TX, United States

**Keywords:** bioengineered alternative tissue, porcine (pig) model, vascular constructs, 3D printed, sterolithography

## Abstract

Recently developed biofabrication technologies are enabling the production of three-dimensional engineered tissues containing vascular networks which can deliver oxygen and nutrients across large tissue volumes. Tissues at this scale show promise for eventual regenerative medicine applications; however, the implantation and integration of these constructs *in vivo* remains poorly studied. Here, we introduce a surgical model for implantation and direct in-line vascular connection of 3D printed hydrogels in a porcine arteriovenous shunt configuration. Utilizing perfusable poly(ethylene glycol) diacrylate (PEGDA) hydrogels fabricated through projection stereolithography, we first optimized the implantation procedure in deceased piglets. Subsequently, we utilized the arteriovenous shunt model to evaluate blood flow through implanted PEGDA hydrogels in non-survivable studies. Connections between the host femoral artery and vein were robust and the patterned vascular channels withstood arterial pressure, permitting blood flow for 6 h. Our study demonstrates rapid prototyping of a biocompatible and perfusable hydrogel that can be implanted *in vivo* as a porcine arteriovenous shunt, suggesting a viable surgical approach for in-line implantation of bioprinted tissues, along with design considerations for future *in vivo* studies. We further envision that this surgical model may be broadly applicable for assessing whether biomaterials optimized for 3D printing and cell function can also withstand vascular cannulation and arterial blood pressure. This provides a crucial step toward generated transplantable engineered organs, demonstrating successful implantation of engineered tissues within host vasculature.

## Introduction

In the current era of solid organ transplantation, several barriers exist for patients with end- organ disease before they can get a life-saving transplant. First and foremost, the need for donor organs exceeds the availability of solid organs for transplantation in the US and worldwide. Nearly 110,000 people are on the waitlist for organ transplantation in the US alone (based on August 2020 data from the Organ Procurement and Transplantation Network). However, less than 40,000 transplantations were performed in 2019 as there were <20,000 donors that year. The demand for viable, transplantable organs sorely outweighs the supply which leads to the use of marginal or extended-criteria organs, unsurprisingly leading to more complications. Generally, those who are sicker with greater wait time are those who receive organ transplants, thereby increasing the risk for more complications. After transplantation, chronic rejection often leads to end-organ disease again. Immunosuppression regimens, a requirement for transplantation, expose the recipient to greater risks of infection, malignancy and overall greater morbidity to stave off rejection. Moreover, these pitfalls of transplantation assume that patients make it to the transplant list in the first place; often, those without social and economic support will not be eligible. Overall, disruptive new ideas will be necessary to treat patients in the future and to overcome the current and significant barriers one faces in the modern era of solid organ transplantation.

Adequate graft vascularization and efficient integration with host circulation are paramount in translating large-scale engineered tissues into clinically relevant therapies (1, 2). Engineering a thick and complex scaffold that can sustain a metabolically active cell population requires a highly organized, built-in vascular network. Although tremendous emphasis has been placed on development of biomaterials to enhance cell survival and integration, comparatively little effort has been directed toward developing direct transplant surgery models for graft implantation (3). Currently, most techniques for implanting pre-vascularized tissue constructs rely on the ingrowth of host vessels and anastomosis at the capillary level for perfusion of the engineered vasculature (4–7). This method of anastomosis has been shown to take days to weeks to perfuse the implanted microvascular network and appears to be highly dependent on the geometry of the microvascular network (7–9). However, for scaffolds larger than a few millimeters this time frame may be too slow to ensure the viability of cells within metabolically active tissues (10).

In the past 10 years, an alternative strategy has begun to emerge, enabled by the development of increasingly sophisticated hardware and materials for additive biomanufacturing. Embedded vascular networks present a more immediate route to achieving blood flow through engineered tissues than the protracted process of microvascular invasion and anastomosis (11). Indeed, an expanding suite of 3D printing technologies encompassing embedded extrusion printing (12, 13), hydrogel stereolithography (14), and sacrificial templating (15–18) now permit the generation of model tissues pervaded by extensive networks of perfusable channels, which can also serve as a substrate for seeding of an endothelial monolayer. In particular, hierarchical vascular trees with a single inlet and outlet have been demonstrated. In contrast to earlier macroporous architectures, single-inlet/single-outlet tissues raise the possibility of directly connecting an implanted tissue to a host artery and vein, in analogy to a conventional organ transplant.

Direct surgical anastomosis has been widely studied in the context of engineered vascular grafts (VG) (19), resulting in many validated materials (20) for this application and well-developed surgical techniques (21). Such grafts, however, typically comprise a tube of a fibrous polymer and inhabit a different regime of mechanical properties compared to typical hydrogels and gel-based tissue constructs. Moreover, VGs focus on bypassing occluded or damaged vessels, and rarely include branches for connections with capillaries or smaller vessel networks that would nourish surrounding tissues. Outside of VGs, studies of direct anastomosis between engineered and native vascular networks are relatively few in number. For example, Peterson and colleagues demonstrated preliminary success implanting engineered lungs *in vivo* for short time periods (22). Lungs were decellularized and seeded with rat lung epithelium and lung microvascular endothelium then cultured for a week in a bioreactor before being implanted *in vivo* for periods up to 2 h. However, there were challenges in maintaining barrier function of small vessels and coverage with endothelial cells. Similarly, Uygun et al. implanted a decellularized and recellularized liver, where the engineered tissue was implanted *in vivo* for up to 8 h before significant clotting occurred (23). In terms of tissue constructs fabricated *de novo*, Zhang et al. have demonstrated the *in vivo* implantation of microfluidic channel networks, fabricated by individual layer fabrication and sequential lamination, in the femoral vessels of a rat, which remained patent for up to 1 week (24). Another example from Hooper and colleagues involves 3D printing of a sacrificial Pluronic F127 Loop which is embedded in a collagen gel, then removed by aqueous dissolution, leaving an open channel (25). The group demonstrated that by using a polyglactin surgical mesh applied to the collagen gel, they could surgically anastomose the channels of their engineered vessel to the femoral artery and vein. These channels remained patent between 15 min to 24 h *in vivo*. Finally, in a previous study, our group implanted silicone tissue mimics with patterned vascular networks in-line in a rat femoral artery graft and found that we could establish and monitor pulsatile blood flow through the constructs (26).

Collectively, these prior studies present an informative starting point for in-line cannulation of engineered vascular networks; yet, as is the case for VGs, the materials employed diverge considerably from those currently investigated for soft tissue biofabrication. Thus, there is little known about how patterned hydrogels can be effectively connected to host vasculature. More broadly, it is not yet clear what material compositions or architectures could simultaneously support cell function, be amenable to 3D printing, and be mechanically robust enough to tolerate vascular cannulation and arterial blood pressure. Crucially, the size of the host vessels which could serve as connection points to an implanted tissue depend on the size of the animal model, as does the volumetric flow rate of blood through the implant. Thus, while rodent studies are beneficial for interrogating the function and integration of engineered tissues, direct cannulation models must also be developed in larger animal models as a bridge to eventual human clinical use.

Here, we introduce a porcine arteriovenous shunt as a surgical model for in-line cannulation of patterned hydrogel materials. We fabricate compliant poly(ethylene glycol) (PEGDA) hydrogels using our previously described projection stereolithography platform (14) and investigate the *in vivo* cannulation of a patterned perfusable vascular channel. In short-term implantation studies, we validate that the gels withstand arterial pressure and support blood flow. This study is an early demonstration of connecting a soft, 3D printed hydrogel in-line with a mammalian vascular supply. Such implantable models open a rich design space where the architectural freedom of additive manufacturing may be unified with the physiologic and therapeutic relevance of *in vivo* implantation.

## Materials and Methods

### Chemical Synthesis

Poly(ethylene glycol) diacrylate (PEGDA; 6 and 35 kDa) was prepared as described in Grigoryan et al. (14). Briefly, poly(ethylene glycol) was reacted with triethylamine and acrylolyl chloride in anhydrous dichloromethane under argon overnight. The percent acrylation was determined to be 99% by H NMR, and yields generally ranged from 80 to 90% for batch sizes up to 350 g. Lithium phenyl-2,4,6-trimethylbenzoylphosphinate (LAP) was prepared, as described previously (27). Dimethyl phenylphosphinite was reacted with 2,3,6-trimethylbenzoyl chloride under argon overnight at room temperature. A 4-molar excess lithium bromide in 2-butanone was added to the mixture, which was then heated to 50°C to allow the formation of a solid precipitate. The mixture was cooled to room temperature for 4 h before being filtered with excess 2-butanone and diethyl ether. Yields up to 90% were achieved for batch sizes up to 30 g. All reagents were purchased from Sigma-Aldrich.

### Design and Fabrication of Hydrogels

**Figure 1:**
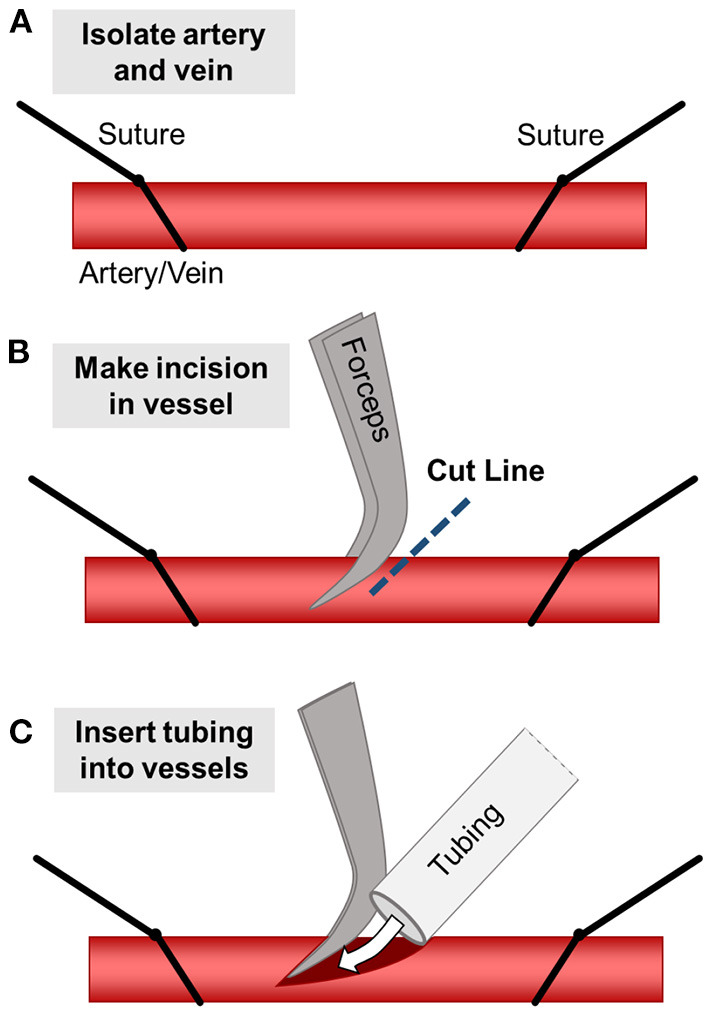
Surgical diagram for inserting constructs into pig models. **(A)** First, the target artery and vein are isolated and controlled using two silk suture loops. **(B)** The sutures are pulled to restrict blood flow and a small incision is made partially through the vessel. **(C)** The tubing (with the vascular construct attached) is inserted into the vessel and clamps are removed, allowing blood to flow through the implanted construct. Tubing is secured within the vessels using silk sutures.

PEGDA hydrogels were fabricated via projection stereolithography using LAP photoinitiator and tartrazine photoabsorber with 405 nm light (30 s exposure at 16.5 mW cm^−2^). We identified a PEGDA formulation containing 20 wt% 6 kDa PEGDA plus 20 wt% 35 kDa PEGDA as optimal for printability and compliant for cannulation. The gels used for these studies were rectangular with outer dimensions of 19 × 30 mm and contain a bulb-shaped, interior channel with a diameter of 1.5 mm (Figure 1). Gels designated for implantation were printed with 100 U/mL heparin added to the print formulation.

To address challenges in securing the Silastic tubing within the PEGDA hydrogels, the hydrogels were housed in 3D printed plastic cases and the tubing was secured to the plastic cases using cyanoacrylate glue. The plastic cases were fabricated with a Fortus 450 MC using the biocompatible polycarbonate filament (PC-ISO, Stratasys) and were autoclaved prior to implantation.

### Burst Pressure Testing

To ensure that the gels could withstand arterial blood pressure, the burst pressure of the hydrogel gel designs was tested prior to implantation. Pressure was applied to the gels using a custom built pneumatic system which is capable of applying pressures up to 30 PSIG (~1,500 mmHg) (https://github.com/MillerLabFTW/OpenSourcePneumaticSystem). First, printed hydrogels were allowed to swell to equilibrium prior to testing. The gels were then secured to a glass slide using cyanoacrylate glue and connected with 26- and 22-gauge catheters. Tubing connected to the venous outlet of the gel was clamped while the arterial end was connected to the pneumatic pressure system. Pressure was slowly increased from 0 to 15 PSI (775 mmHg) and recorded using a digital pressure gauge at the point of channel rupture. Gels were tested in the PC cases and catheterized with Silastic tubing to mimic the conditions *in vivo*.

### Surgical Implantation

**Figure 2:**
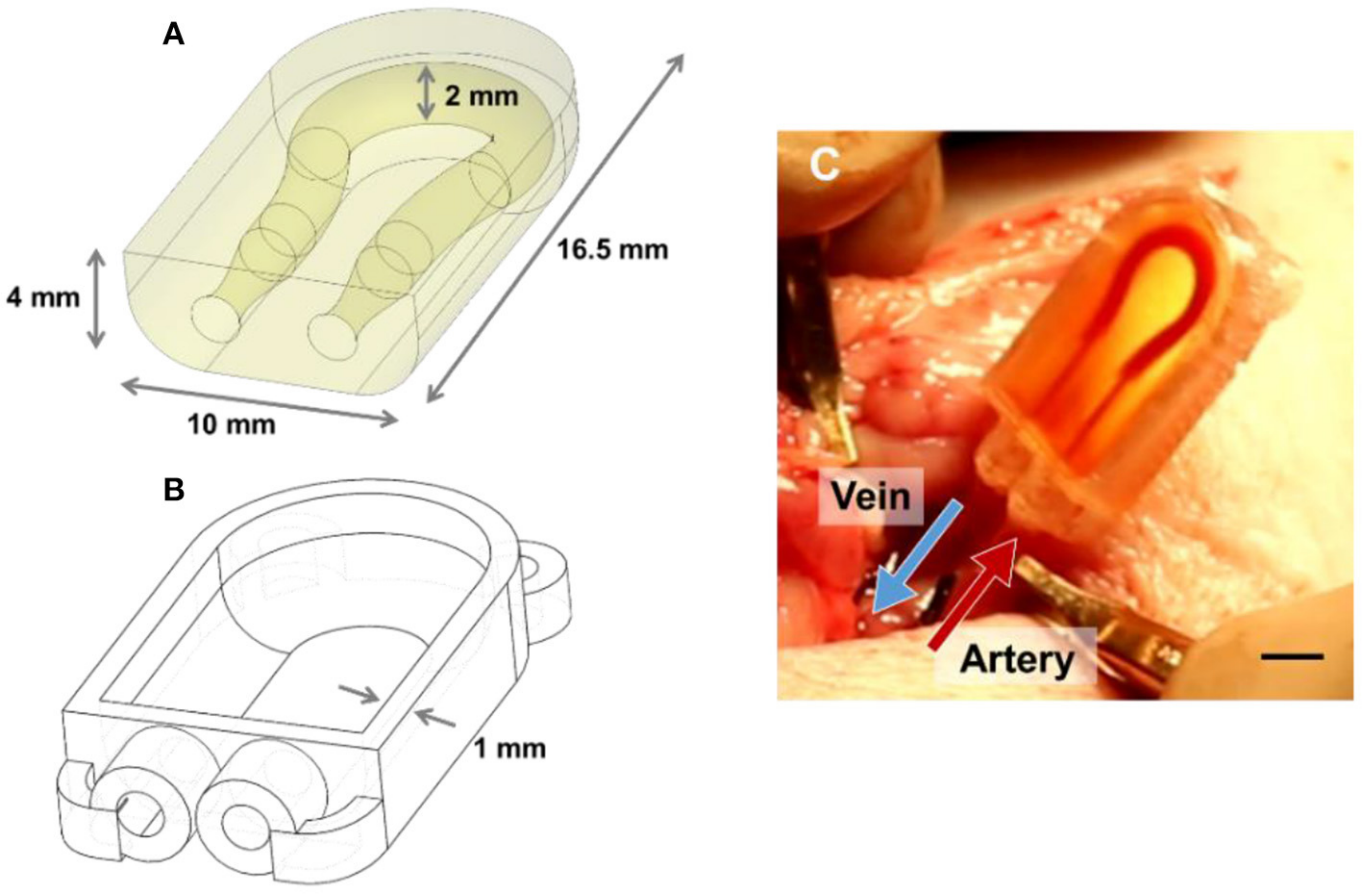
Schematic of PEGDA AV vascular shunt used for implantation in porcine model. **(A)** Schematic of the modified hydrogel use for implantation in the neck of 5–10 kg piglets. **(B)** Schematic of 3D printed PC case for hydrogel geometry in **(A)**. **(C)** Image of a gel implanted as an AV shunt linking the carotid artery to the jugular vein. Scale bar = 5 mm.

During implantation *in vivo*, we initially opted for the femoral vascular bundle but found that *in vivo* survival studies would require significant limitations in the movement of the piglets. Therefore, we focused on implantation in the neck, which would allow for free-range movement of the piglet within their housing to better illustrate the clinical applications and durability of the model. An incision was made over the carotid vascular bundle, and the soft tissue was dissected down to the carotid artery and internal jugular vein. These vessels were circumferentially isolated, and proximal and distal control were obtained. The vessels were clamped proximally and distally to restrict blood flow, after which an arteriotomy was created and the silastic tubing attached to the hydrogel construct was cannulated into the vessel. This was secured in place with a silk suture. A similar incision was made along the femoral vein, and the other end of the silastic tubing was used to cannulate the vein, creating an arteriovenous configuration with the hydrogel construct, as shown in Figure 2. Tubing is then secured using silk sutures and the clamps are released to allow blood flow through the vascular construct.

### Ultrasound and Imaging

During the final round of implantations, a GE Vivid 7 Ultrasound imaging system was used to measure flow using color Doppler trans-dermally following implantation. The Doppler ultrasound allows for real-time assessment of blood flow within vasculature, in a non-invasive manner. In this case, the ultrasound probe was laid directly over the hydrogel constructs to prove flow using color Doppler to assess the direction of blood flow through the vessels, proximal to the construct, within the construct, and distal to the vascular construct.

### Histology for *in vivo* Samples

Two different methods were used for histological analysis of the hydrogels: vibratome sectioning in which embedding of the samples is not required, and resin embedding using an ultramicrotome for sectioning. All samples were fixed in 4% paraformaldehyde overnight, and then washed several times in PBS before sectioning or embedding. In sectioning via vibratome, sectioning was conducted using a VT 1000S vibrating microtome (Leica) to generate 500 μm thick sections with the speed and frequency set to 3 and 9, respectively. Embedding using the JB4 resin (Polysciences) was done as described previously (28). The gel samples were cut into smaller pieces before being placed in the JB4 infiltration solution until the samples sunk to the bottom of the tube. The infiltration solution was then changed, incubated at room temperature for a few hours, replaced once again for overnight incubation at room temperature. After infiltration, the sections of gels were placed in molds, which were then filled with the JB4 embedding solution and allowed to harden overnight under argon. Sectioning of the resin-embedded samples was conducted using an EM UC7 Ultramicrotome (Leica) in which sections were cut at 6–8 μm thick using a glass knife.

Hoechst stain was used to identify nuclear material in both JB4-embedded and vibratome-sectioned samples. Hoechst 6024 (Sigma) was added to sections at a concentration of 0.5 μg/mL and allowed to incubate for 20 min at room temperature before being rinsed several times using DI water. Toluidine blue stain is a basic thiazine metachromatic dye commonly used as an alternative to H&E staining, where tissue and cell components will stain violet to red/yellow. Toluidine blue was used only for JB4 embedded samples. Toluidine blue stain was mixed using 5 g sodium borate, 2.5 g toluidine blue (Sigma Aldrich) and 500 mL DI water. Slides containing resin-embedded sections were placed on a hot plate at 100–150°C for at least 30 min prior to staining. The sections were then stained for 1–2 s before being rinsed with DI water several times. Slides were allowed to dry on hot plate for 5–10 min before being covered with a coverslip.

### Animal Care and Safety

All animal care and surgical procedures are performed according to Protocol AN-7216 as approved by the Institutional Animal Care and Use Committee (IACUC) of Baylor College of Medicine and secondarily approved by Rice University IACUC. Our veterinarian was present for study design, all experiments, and oversaw all animal care. The Center of Comparative Medicine at Baylor College of Medicine was also consulted to provide anesthesia for these animal studies and also to serve as a second veterinarian on-site to ensure all animal studies were humane and in accordance with IACUC protocol.

## Results and Discussion

### Design of Vascular Conduits for Porcine Implantation

Our prior implantation studies in rat femoral arteries utilized straight-channel or branched ladder designs in a surgical model where the construct was inserted in-line into the artery (26). Here, we transitioned toward an arteriovenous (AV) shunt model which connects the vein to the artery. This change in geometry helped maintain the fluidic connection between the hydrogel and the catheters during leg movement, which is a key challenge following *in vivo* implantation of vascularized constructs. Additionally, we hypothesized that the greater pressure differential between the artery and the vein (as compared to the proximal vs. distal artery) would aid in increasing blood flow rate and thereby help prevent clotting (29, 30). Compared to prior rat studies, this gel design also had to be adapted for larger vessel diameter of the 5–10 kg piglets and the different anatomy of the surgical site. Channel diameters were increased to accommodate 5 French Silastic tubing (0.76 mm ID, 1.65 mm OD; Silastic). The inlets and outlets of the channels are tapered to encourage a tight seal between the gel and the inserted tubing. Finally, the PEGDA hydrogel was placed within a PC case to help secure the tubing in place within the gel following wound closure (Figure 2B).

Burst pressure for the updated gel design was 325.8 ± 29.3 mmHg (*N* = 5), with a minimum burst pressure of 305.6 mmHg. The gel geometry used for porcine models had a lower burst pressure than gels implanted in rats, likely due to the slimmer profile of these gels relative to the channel diameter. This reduced profile was implemented due to the restricted space within the implantation site; however, the burst pressure of the constructs is still well above systemic blood pressure of piglets at ~120 mmHg (31).

### *In vivo* Implantation of Constructs in Porcine Model

In designing experiments, we planned for 4 cohorts of study to enable consistency and reproducibility while allowing for fabrication changes to the hydrogel, adjustments in surgical technique and flow measurement and accommodating the practicalities of animal care. A full summary of each experimental design and the results thereof is provided as Table 1.

**Table 1:** Summary of all surgeries performed on porcine model.

**Study number**	**Experimental design**	**Results**	**Other notes**
1.1	•Non-survival, monitor under anesthesia •Implant femoral AV shunt	•Construct and tubing were connected using surgical mesh and cyanoacrylate glue •Connection between construct and tubing was not secure enough •Pliability of hydrogel made implantation difficult	Surgery was not successful
1.2	•Non-survival, monitor under anesthesia •Implant femoral AV shunt	•PLA case was introduced to secure tubing to construct •Low signal quality from the flow probe, but flow was measured between 18 and 10 mL/min and confirmed upon removal of construct after ~2 h	Flow detected after 2 h, but inconsistent signal from flow probe
1.3	•Non-survival, monitor under anesthesia •Implant femoral AV shunt (L) and AA (R)	•Constructs with PLA case implanted in L and R leg •Flow was confirmed in left leg after 2 h, but damage to vessels in right leg potentially halted flow on R side	Flow detected after 2 h by flow probe
1.4	•Non-survival, monitor under anesthesia •Implant femoral AV shunt (L) and AA (R)	•Constructs implanted in L and R leg •Flow was confirmed through both constructs after 2 h, though gel in R leg had some clot	Flow detected after 2 h by flow probe
2.1	•Survival study •Recovery after 8 h •Implant femoral AV shunt (L)	•Smaller gel design fit better beneath skin without inhibiting implantation procedure •Added bead at the end of tubing allowed us to reduce the total length of tubing inserted into the vessels •Pig did not recover from anesthesia, thus euthanized •No apparent damage to the construct after removal	Flow detected upon implantation by flow probe, piglet did not recover from anesthesia
2.2	•Survival study •Recovery after 8 h •Implant femoral AV shunt (L)	•Surgery was successful, though signal from the flow probe was inconsistent •After ~6 h the construct was removed, found tubing to be kinked. Blood in the channel of the construct was completely clotted	Flow not detected by flow probe after 6 h, kink in the tubing identified with occlusive clot
3.1	•Survival study •Recovery after 6 h •Implant AV shunt (L) in neck	•Fit between tubing and new case material was difficult •Eventually implanted a gel successfully, closed wound and recovered animal •After some initial bleeding, no apparent issues, despite low readings from flow probe •Construct was removed ~6 h after implantation, fully clotted	Flow not detected by flow probe after 6 h, occlusive clot identified in the tubing
3.2	•Survival study •Recovery after 6 h •Implant AV shunt (L) in neck	•Fit between tubing and new case material was difficult •Eventually implanted a gel successfully without leaks, closed wound and recovered animal •Construct dislodged shortly after recovery and animal was euthanized	Surgery was not successful, construct dislodged
4.1	•Survival study •Recovery after 5 h •Implant AV shunt (L) in neck	•Hydrogel, case, and tubing were assembled prior to implantation and tubing was secured in place using cyanoacrylate glue •The piglet recovered after implantation and monitored for ~5 h prior to euthanasia	Flow detected by duplex after 5 h, and the construct appeared patent upon removal
4.2	•Survival study •Recovery after 5 h •Implant AV shunt (L) in neck	•Hydrogel, case, and tubing were assembled prior to implantation and tubing was secured in place using cyanoacrylate glue •The piglet recovered after implantation and monitored for ~5 h prior to euthanasia	Flow detected by duplex after 5 h, and the construct appeared partially clotted upon removal
4.3	•Survival study •Recovery after 5 h •Implant AV shunt (L) in neck	•Hydrogel, case, and tubing were assembled prior to implantation and tubing was secured in place using cyanoacrylate glue •The piglet recovered after implantation and monitored for ~5 h prior to euthanasia	No flow was detected after 5 h, and the construct was clotted upon removal
4.4	•Survival study •Recovery after 5 h •Implant AV shunt (L) in neck	•Hydrogel, case, and tubing were assembled prior to implantation and tubing was secured in place using cyanoacrylate glue •The piglet recovered after implantation and monitored for ~5 h prior to euthanasia	Flow detected by duplex after 5 h, and the construct appeared patent upon removal

*AV, arteriovenous; AA, arterio-arterial; R, right; L, left*.

Cohort 1 included four non-survival implantations over the course of 2 operative days to assess the feasibility of implanting pliable hydrogels in the groin of the porcine model, ensuring size matching was reasonable, and evaluating the modality by which to measure blood flow *in vivo*. The piglets were anesthetized for 2 h, during which time the hydrogels were implanted in the groin and flow was assessed using an in-line flow probe. We found that the hydrogels indeed allowed for blood flow using the model's blood pressure to sustain flow. However, handling the hydrogel and keeping the tubing in place within the gel and in the blood vessel was a considerable design challenge. To address these challenges, we developed a 3D printed plastic case to sheathe the hydrogel and hold the tubing in place, allowing easier implantation within the femoral artery and vein or femoral artery to distal femoral artery. Furthermore, we found that the flow probe was unreliable and difficult to place along the distal outflow tract, and so assessing flow proved inconsistent. Three of the 4 piglets showed continued blood flow through the hydrogel construct at the end of the 2 h. In particular, two piglets received dual femoral implants with the left construct in arteriovenous (AV) configuration and the right as arterio-arterial (AA) configuration; in these models; the AV implants demonstrated qualitatively less clotting and better patency.

For Cohort 2 we began the survival studies, wherein the fabricated hydrogel construct was implanted under anesthesia in AV configuration. After piglets recovered from anesthesia, they could ambulate for 6–8 h while blood flow was assessed prior to euthanasia and recovery of the construct post-mortem. The first study subject never recovered from anesthesia, and thus was euthanized after successful implantation. The vascular construct of the second piglet was found kinked at the venous outlet, and thus clotted completely after 6 h at explantation, but proved that the pig can survive implantation of the construct, recover from anesthesia, and ambulate without dislodging the construct.

For Cohort 3, we determined that implantation in the neck is preferable to the femoral groin, as it allows for greater surgical exposure and reduced movement of the construct following recovery from anesthesia, which was hypothesized to obviate the issue of kinked tubing. Unfortunately, this cohort was complicated by a construct that dislodged, requiring immediate euthanasia of one piglet. The other pig underwent successful implantation though no flow was observed upon explantation, explained by an occlusive clot within the construct. Flow probe measurements had large variations and proved unreliable. From this cohort we learned to utilize ultrasound with duplex to assess flow upon implantation and prior to explantation, which was found to be easier to utilize and reproduce.

**Figure 3:**
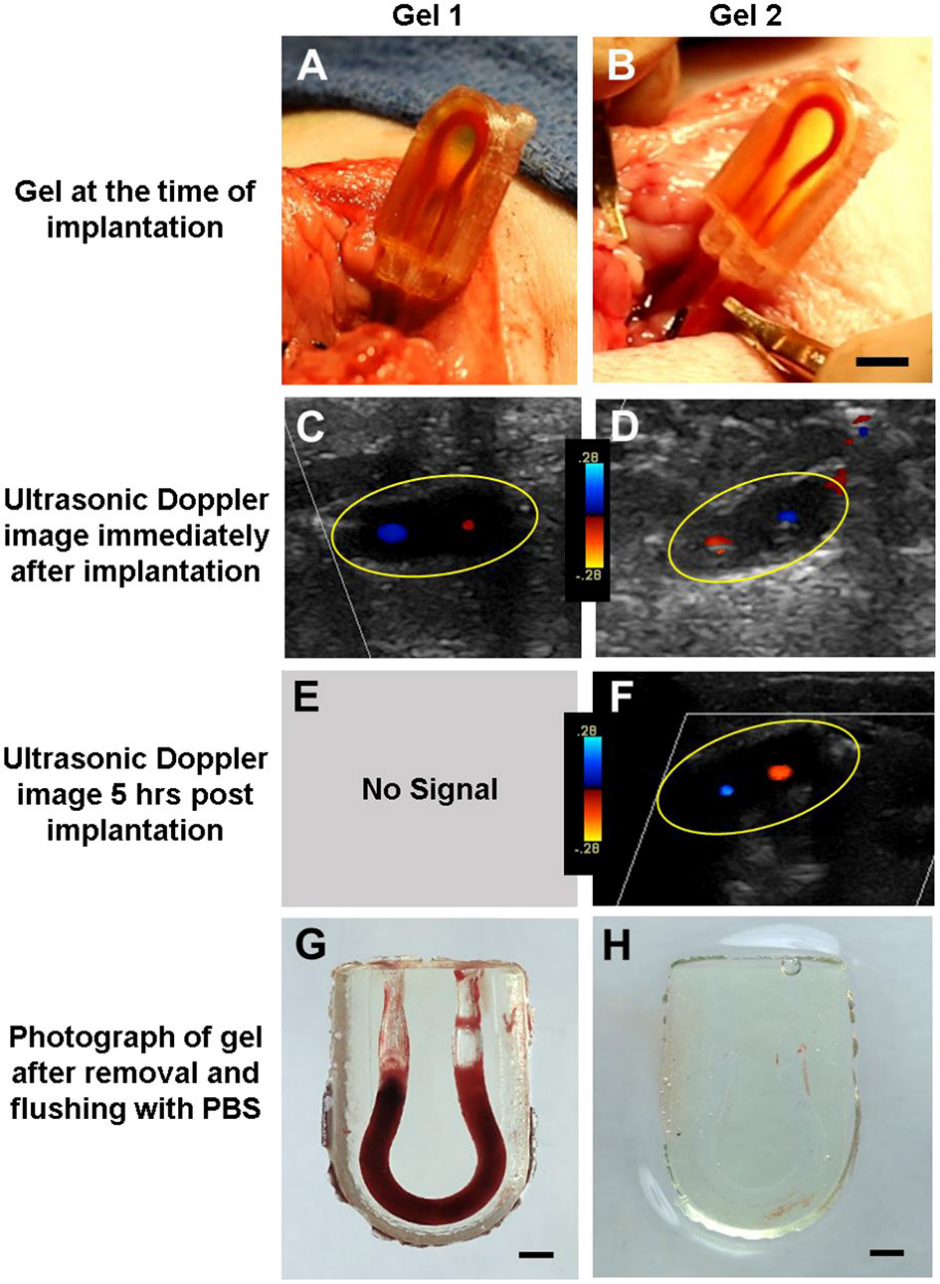
Assessment of PEGDA hydrogels implanted in a porcine model. **(A,B)** Image of gel at time of implantation. Scale bar = 5 mm. **(C,D)** Ultrasonic Doppler image of gel immediately after wound closure, displaying flow through both channels. **(E,F)** Ultrasonic Doppler image 5 h after implantation, where no signal was detected for Gel 1. **(G,H)** Images of gel after explant and flushing with saline. Saline flush dislodges clots which form postmortem but preserve more stable clots seen in a few areas of **(H)**. Scale bar = 2 mm. Gels 1 and 2 in this figure correspond to conditions 4.3 and 4.4 in Table 1, respectively.

Cohort 4 was the most successful, with three of the four subjects maintaining flow throughout the entire survival study—from implantation, recovery after anesthesia, and ambulation for 5 h all the way to just before euthanasia and explantation. In these trials, the ultrasound probe was laid directly over the hydrogel constructs, allowing us to measure flow using color Doppler to assess the direction of blood flow through the vessels, proximal to the construct, within the construct, and distal to the vascular construct (Figure 3). When implanted *in vivo*, the PC cases and cyanoacrylate glue prevented the tubing from being dislodged from the hydrogels as the piglets moved about freely, and the PEGDA hydrogels were capable of withstanding systolic pressures. With modifications to the placement of the construct and the type of casing used to house it, we were able to improve our surgical technique and handling of the blood vessels, which likely enabled sustained blood flow with less thrombosis in our explants.

These experiments demonstrate that soft PEGDA hydrogels fabricated via projection stereolithography can sustain blood flow *in vivo* within the porcine model over hours. Additionally, these studies uncovered key design criteria required for *in vivo* anastomosis of hydrogels that are not often considered when designing vascularized tissues. Young pigs were required for this protocol as they closely resemble human anatomy in design and size, and serves as a comparable model necessary for a gradual and stepwise increase in caliber of the vasculature with which we work. This is especially pertinent as our work may eventually be translated to the clinical setting, in both the adult and pediatric populations. As we aim to study blood flow through engineered vasculature with the goal of using these vessels to perfuse 3D printed organs for transplantation, we acknowledge the limitations presented by these cohorts. These were a small number of cases, and each cohort had thrombotic and/or bleeding events. More experiments will be necessary to address the issue of clotting, and tease out whether surgical technique was responsible or a hypercoagulable response to the hydrogel, or a combination of both. Lengthening the time in which the piglets are observed with the hydrogels in place will also help elucidate the tendency to clot.

### Histology for *in vivo* Samples

**Figure 4:**
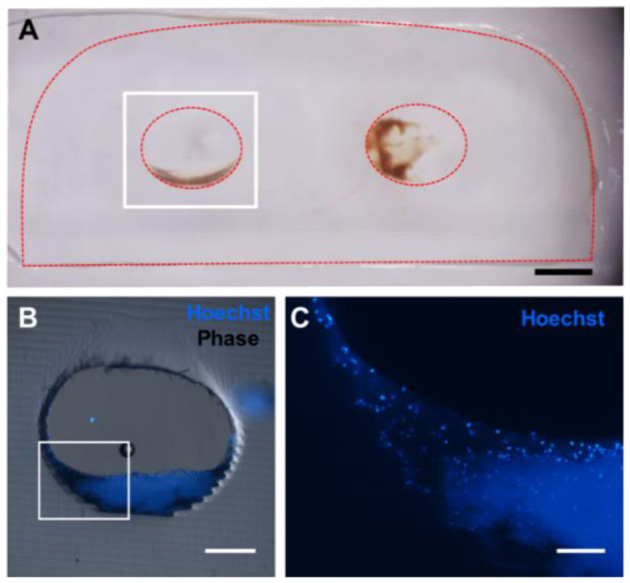
Five hundred micrometer thick vibratome sections of gels implanted for 6 h *in vivo*. **(A)** Reflected light color photo of a 500 μm thick vibratome section, where the red dotted line indicates the edges of the gel. Scale bar = 1 mm. **(B)** Phase contrast/Hoechst overlay of Hoechst-stained channel, outlined in white in **(A)**. Scale bar = 250 μm. **(C)** Zoomed in view of **(B)** showing individual nuclei. Scale bar = 100 μm.

Though cryosectioning was initially planned for histological analysis of implanted gels, the dense concentration of high molecular weight PEGDA chains made this formulation incompatible with traditional cryosectioning, despite the screening of different embedding and freezing techniques. Therefore, we turned to alternative approaches for sectioning our implanted hydrogels: vibratome sectioning at 500 μm and using an ultra-microtome to section samples embedded in JB4 resin. Initial results revealed the presence of nucleated cells within well-formed clots (Figure 4). Additionally, in thinner resin-embedded samples, toluidine blue highlighted the cells present at the edge of the gel. These histological techniques should also be used in future studies to assess protein adsorption, cell adhesion, and the timing of any clotting that occurs.

**Figure 5:**
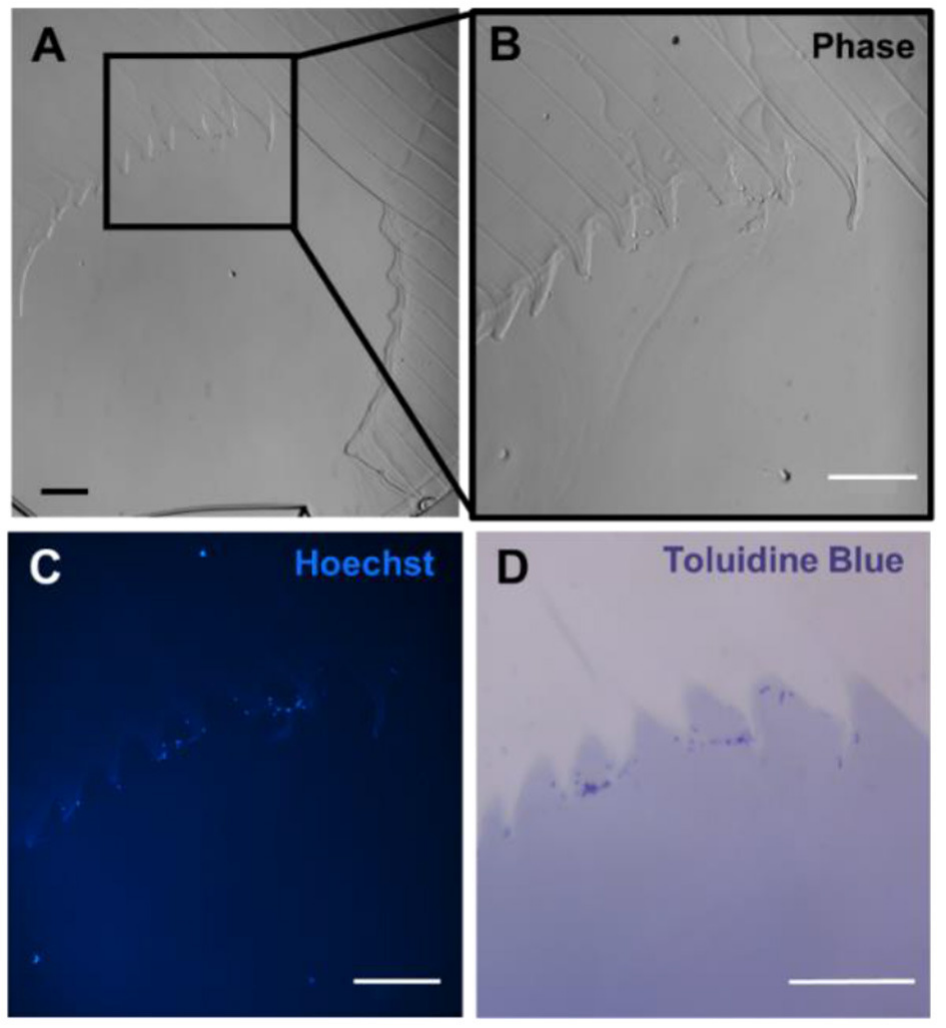
Resin-based histology of gels implanted in porcine model. **(A)** Phase contrast image of gel channel, where section is 6 μm thick. **(B)** Zoomed image of the channel showing the presence of cells adhering to the channel wall. **(C)** Hoechst stain of the gel demonstrated nucleated cells along the wall. **(D)** Toluidine blue stain highlighting cell material in dark purple. Scale bars = 100 μm.

These developments in the sectioning of highly elastic hydrogels allowed for us to further assess the root causes of clotting events. Figure 5 suggests that surface roughness with hydrogel channels can affect clotting. To test this hypothesis constructs could be fabricated using smaller layer heights at 25 μm or rotate the printing orientation of the hydrogels, which would alter the location of any “stair stepping” effect within the channels during printing. For future work, we are interested in exploring the role of channel surface topology and surface chemistry on clotting dynamics. With this in mind, histological analysis could be used to assess the formation of early clots within the channels relative to the geometry of the channel surface. Though PEG is typically considered to be bio-inert and resistant to protein adsorption, previous groups have demonstrated that proteins indeed adsorb to PEG hydrogels implanted *in vivo* (32). Swartzlander and colleagues used a proteomic analysis to demonstrate that the addition of cell adhesive ligands to PEG-based hydrogels can decrease the foreign body response *in vivo*. In future work, similar techniques could be applied to study the adsorption rate of different proteins to PEG-based hydrogels *in vitro*. The influence of different PEGDA formulations, different exposure times, and different surface treatments on protein adsorption could assist in preventing clotting *in vivo*. More generally, the native endothelium is widely understood to prevent and modulate the thrombosis cascade (33, 34). Many demonstrations now exist of endothelial cell seeding within 3D printed vascular channels (15–17), and we expect that endothelialized implants would exhibit prolonged patency and fewer clots as compared to the preliminary acellular gels investigated here. It is important also to note clots found in porcine models which showed flow on US likely represent postmortem clotting, as flow was established prior to euthanasia.

Ultimately, this study demonstrates the development of a surgical technique for implanting 3D printed soft hydrogels *in vivo* with direct anastomosis to host vasculature. Furthermore, we have shown that these hydrogels can sustain blood flow *in vivo* with the porcine model's own blood pressure over 5 h and that Doppler ultrasonography can reliably assess flow proximal to the vascular construct, within it, and distal to the construct over time. The histology of these vascular hydrogels imply high MW hydrogels could be an efficient method to incorporate cell-adhesive ligands onto the surface of the hydrogels for future studies focusing on minimizing clot formation *in vivo* or otherwise manipulating the interactions between circulating cells and the implanted hydrogel.

## Conclusion

During these studies we designed, fabricated, and tested a 3D printed soft hydrogel compatible with direct anastomosis to host vasculature *in vivo*. These results demonstrate that our pSLA fabrication approach and newly developed surgical technique are sufficient for producing vascular shunts that are compatible with surgical anastomosis in piglets. In addition, the flexibility of our pSLA fabrication system, with respect to channel geometry and materials, allows us to iteratively address challenges with clotting or surgical implantation related to gel architecture or materials. This work helped uncover key design criteria for the *in vivo* anastomosis of engineered tissues that are not often considered by researchers. We expect that the porcine AV shunt model introduced here will open the design space for exploring the *in vivo* implantation and integration of engineered regenerative tissues containing patterned vascular networks.

## Data Availability Statement

The original contributions generated for the study are included in the article/supplementary material, further inquiries can be directed to the corresponding author/s.

## Ethics Statement

In compliance with ethical standards and standards of research involving animals, this animal study was reviewed and approved by both the Baylor College of Medicine IACUC Committee and the Rice University IACUC Committee.

## Author Contributions

NG and SP contributed to the design and implementation of the research, to the analysis of the results, and to the writing of the manuscript. JCM, ID, and DY contributed to design and implementation of the research. BG and JSM contributed to the design and analysis. IK contributed to the design and writing of the manuscript. All authors contributed to the article and approved the submitted version.

## Conflict of Interest

JSM and BG are cofounders of and hold an equity stake in the startup company Volumetric, Inc. The remaining authors declare that the research was conducted in the absence of any commercial or financial relationships that could be construed as a potential conflict of interest.
